# 3-[(*E*)-2-Chloro-3,3,3-trifluoro­prop-1-en-1-yl]-*N*-(2-fluoro­phen­yl)-2,2-dimethyl­cyclo­propane-1-carboxamide

**DOI:** 10.1107/S1600536810050464

**Published:** 2010-12-11

**Authors:** Fan-Yong Yan, Dong-Qing Liu, Jing-Yun Wen, Yun-Ying Gao, Ai-Mei Li

**Affiliations:** a Key Laboratoy of Hollow Fiber Membrane Materials & Membrane Processes, School of Environmental & Chemical Engineering, Tianjin Polytechnic University, Tianjin 300160, People’s Republic of China; b School of Materials Science and Engineering, Tianjin Polytechnic University, Tianjin 300160, People’s Republic of China; c School of Environmental & Chemical Engineering, Tianjin Polytechnic University, Tianjin 300160, People’s Republic of China

## Abstract

The phenyl ring in the title compound, C_15_H_14_ClF_4_NO, makes a dihedral angle of 80.3 (3)° with the cyclo­propane ring. In the crystal, mol­ecules are linked by N—H⋯O hydrogen bonds into chains running along the *a* axis.

## Related literature

The title compound is an inter­mediate for tefluthrin, an insecticide controlling a wide range of soil insect pests, see: Punja (1981 ▶). For the synthesis of the title compound, see: Liu & Yan (2007 ▶).
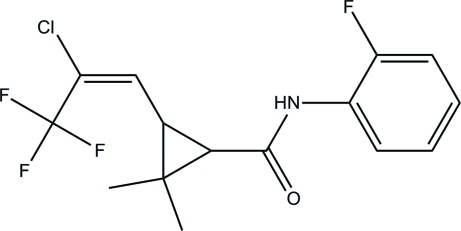


## Experimental

### 

#### Crystal data

C_15_H_14_ClF_4_NO
*M* *_r_* = 335.72
Orthorhombic, 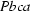

*a* = 9.4604 (19) Å
*b* = 17.785 (4) Å
*c* = 18.879 (4) Å
*V* = 3176.6 (11) Å^3^
*Z* = 8
Mo *K*α radiation
μ = 0.28 mm^−1^
*T* = 293 K
0.40 × 0.06 × 0.06 mm

#### Data collection

Rigaku Saturn CCD area-detector diffractometer
Absorption correction: multi-scan (*CrystalClear*; Rigaku/MSC, 2005 ▶) *T*_min_ = 0.896, *T*_max_ = 0.983
28794 measured reflections
3488 independent reflections
2848 reflections with *I* > 2σ(*I*)
*R*_int_ = 0.047

#### Refinement

*R*[*F* ^2^ > 2σ(*F* ^2^)] = 0.049
*wR*(*F* ^2^) = 0.135
*S* = 1.07
3488 reflections
206 parameters
H atoms treated by a mixture of independent and constrained refinement
Δρ_max_ = 0.29 e Å^−3^
Δρ_min_ = −0.37 e Å^−3^

### 

Data collection: *CrystalClear* (Rigaku/MSC, 2005 ▶); cell refinement: *CrystalClear*; data reduction: *CrystalStructure* (Rigaku/MSC, 2005 ▶); program(s) used to solve structure: *SHELXS97* (Sheldrick, 2008 ▶); program(s) used to refine structure: *SHELXL97* (Sheldrick, 2008 ▶); molecular graphics: *SHELXTL* (Sheldrick, 2008 ▶); software used to prepare material for publication: *SHELXTL*.

## Supplementary Material

Crystal structure: contains datablocks I, global. DOI: 10.1107/S1600536810050464/bt5406sup1.cif

Structure factors: contains datablocks I. DOI: 10.1107/S1600536810050464/bt5406Isup2.hkl

Additional supplementary materials:  crystallographic information; 3D view; checkCIF report

## Figures and Tables

**Table 1:** Hydrogen-bond geometry (Å, °)

*D*—H⋯*A*	*D*—H	H⋯*A*	*D*⋯*A*	*D*—H⋯*A*
N1—H1⋯O1^i^	0.84 (2)	2.13 (2)	2.9549 (18)	167.3 (18)

Symmetry code: (i) 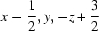.

**Fig. 1.**
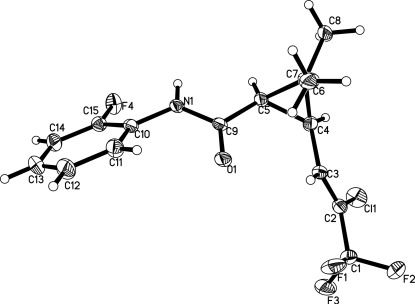
The molecular structure of the title compound drawn with 30% probability ellipsoids. H atoms are drawn as spheres of arbitrary radius.

## supplementary crystallographic information

### Comment

3-((*E*)-2-chloro-3,3,3-trifluoroprop-1-enyl)-2,2-dimethyl cyclopropanecarboxylic acid is a very important intermediate for tefluthrin, an important insecticide controlling a wide range of soil insect pests in maize,sugar beet, and other crops (Punja, 1981). 2-fluoroaniline is also a good structure which has bioactivity. The structure in this article containing both of two active parts may be show some insecticide activity. The present X-ray crystal structure analysis was undertaken in order to study the stereochemistry and crystal packing of the title compound. The dihedral angle between the phenyl ring  and the cyclopropane ring is 99.7 (3)°. In the crystal, the molecules are linked by N—H···O hydrogen bonds to chains running along the a-axis.

### Experimental

The title compound was prepared according to the method of Liu & Yan (2007). The product was recrystallized from methanol and ethyl acetate (8:1) over 2 d at ambient temperature, gave colourless single crystals.

### Refinement

H atoms bonded to C were positioned geometrically with C—H = 0.93–0.98 Å and refined using riding model with *U*_iso_(H) = 1.2U_eq_(C). The H atom bonded to N was located in a difference map and freely refined.

### Crystal data

**Table:**

C_15_H_14_ClF_4_NO	*D*_x_ = 1.404 Mg m^−^^3^
*M**_r_* = 335.72	Melting point: 426 K
Orthorhombic, *P**b**c**a*	Mo *K*α radiation, λ = 0.71073 Å
*a* = 9.4604 (19) Å	Cell parameters from 7273 reflections
*b* = 17.785 (4) Å	θ = 1.6–27.1°
*c* = 18.879 (4) Å	µ = 0.28 mm^−^^1^
*V* = 3176.6 (11) Å^3^	*T* = 293 K
*Z* = 8	Block, colorles
*F*(000) = 1376	0.40 × 0.06 × 0.06 mm

### Data collection

**Table:**

Rigaku Saturn CCD area-detector diffractometer	3488 independent reflections
Radiation source: rotating anode	2848 reflections with *I* > 2σ(*I*)
multilayer	*R*_int_ = 0.047
Detector resolution: 7.31 pixels mm^-1^	θ_max_ = 27.1°, θ_min_ = 2.3°
ω and φ scans	*h* = −12→11
Absorption correction: multi-scan (*CrystalClear*; Rigaku/MSC, 2005)	*k* = −22→22
*T*_min_ = 0.896, *T*_max_ = 0.983	*l* = −24→24
28794 measured reflections	

### Refinement

**Table:**

Refinement on *F*^2^	Secondary atom site location: difference Fourier map
Least-squares matrix: full	Hydrogen site location: inferred from neighbouring sites
*R*[*F*^2^ > 2σ(*F*^2^)] = 0.049	H atoms treated by a mixture of independent and constrained refinement
*wR*(*F*^2^) = 0.135	*w* = 1/[σ^2^(*F*_o_^2^) + (0.076*P*)^2^ + 0.4356*P*] where *P* = (*F*_o_^2^ + 2*F*_c_^2^)/3
*S* = 1.07	(Δ/σ)_max_ = 0.002
3488 reflections	Δρ_max_ = 0.29 e Å^−^^3^
206 parameters	Δρ_min_ = −0.37 e Å^−^^3^
0 restraints	Extinction correction: *SHELXL97* (Sheldrick, 2008), Fc^*^=kFc[1+0.001xFc^2^λ^3^/sin(2θ)]^-1/4^
Primary atom site location: structure-invariant direct methods	Extinction coefficient: 0.026 (2)

### Special details

**Table:**

Geometry. All e.s.d.'s (except the e.s.d. in the dihedral angle between two l.s. planes) are estimated using the full covariance matrix. The cell e.s.d.'s are taken into account individually in the estimation of e.s.d.'s in distances, angles and torsion angles; correlations between e.s.d.'s in cell parameters are only used when they are defined by crystal symmetry. An approximate (isotropic) treatment of cell e.s.d.'s is used for estimating e.s.d.'s involving l.s. planes.
Refinement. Refinement of *F*^2^ against ALL reflections. The weighted *R*-factor *wR* and goodness of fit *S* are based on *F*^2^, conventional *R*-factors *R* are based on *F*, with *F* set to zero for negative *F*^2^. The threshold expression of *F*^2^ > σ(*F*^2^) is used only for calculating *R*-factors(gt) *etc*. and is not relevant to the choice of reflections for refinement. *R*-factors based on *F*^2^ are statistically about twice as large as those based on *F*, and *R*- factors based on ALL data will be even larger.

### Fractional atomic coordinates and isotropic or equivalent isotropic displacement parameters (Å^2^)

**Table:**

	*x*	*y*	*z*	*U*_iso_*/*U*_eq_	
Cl1	0.66430 (6)	0.02672 (3)	0.65669 (3)	0.0561 (2)	
F1	0.96844 (11)	0.16902 (7)	0.62274 (8)	0.0577 (4)	
F2	0.94356 (13)	0.05810 (8)	0.58102 (6)	0.0598 (4)	
F3	0.96350 (13)	0.07416 (8)	0.69269 (7)	0.0635 (4)	
F4	0.40366 (12)	0.33270 (7)	0.91926 (5)	0.0452 (3)	
O1	0.68587 (11)	0.31135 (8)	0.71538 (6)	0.0373 (3)	
N1	0.49478 (14)	0.33592 (8)	0.78429 (7)	0.0282 (3)	
C1	0.9052 (2)	0.10313 (12)	0.63426 (10)	0.0401 (4)	
C2	0.74897 (19)	0.11120 (10)	0.64077 (8)	0.0337 (4)	
C3	0.68226 (17)	0.17637 (11)	0.63525 (9)	0.0328 (4)	
H3	0.7366	0.2189	0.6261	0.039*	
C4	0.52849 (18)	0.18657 (11)	0.64248 (9)	0.0386 (4)	
H4	0.4742	0.1396	0.6430	0.046*	
C5	0.46665 (16)	0.24939 (10)	0.68944 (9)	0.0341 (4)	
H5	0.3798	0.2355	0.7145	0.041*	
C6	0.45206 (18)	0.25234 (12)	0.60950 (9)	0.0430 (5)	
C7	0.5307 (2)	0.31065 (14)	0.56767 (10)	0.0548 (6)	
H7A	0.6195	0.3211	0.5903	0.082*	
H7B	0.4756	0.3559	0.5654	0.082*	
H7C	0.5474	0.2923	0.5206	0.082*	
C8	0.3042 (2)	0.23521 (19)	0.58235 (12)	0.0703 (9)	
H8A	0.2492	0.2806	0.5819	0.105*	
H8B	0.2600	0.1990	0.6128	0.105*	
H8C	0.3101	0.2153	0.5352	0.105*	
C9	0.56086 (16)	0.30093 (10)	0.72953 (8)	0.0282 (4)	
C10	0.56229 (16)	0.38155 (10)	0.83544 (8)	0.0285 (4)	
C11	0.67353 (19)	0.42969 (11)	0.82061 (11)	0.0412 (5)	
H11	0.7070	0.4341	0.7745	0.049*	
C12	0.7352 (2)	0.47147 (13)	0.87490 (12)	0.0551 (6)	
H12	0.8107	0.5032	0.8648	0.066*	
C13	0.6856 (2)	0.46631 (14)	0.94352 (12)	0.0549 (6)	
H13	0.7279	0.4943	0.9793	0.066*	
C14	0.5738 (2)	0.41977 (11)	0.95893 (10)	0.0442 (5)	
H14	0.5390	0.4162	1.0049	0.053*	
C15	0.51453 (18)	0.37860 (10)	0.90486 (9)	0.0324 (4)	
H1	0.409 (2)	0.3269 (10)	0.7911 (10)	0.035 (5)*	

### Atomic displacement parameters (Å^2^)

**Table:**

	*U*^11^	*U*^22^	*U*^33^	*U*^12^	*U*^13^	*U*^23^
Cl1	0.0589 (4)	0.0450 (3)	0.0642 (4)	−0.0103 (2)	−0.0033 (2)	0.0082 (2)
F1	0.0238 (6)	0.0608 (8)	0.0884 (9)	0.0032 (5)	0.0063 (6)	0.0006 (7)
F2	0.0488 (7)	0.0748 (9)	0.0558 (8)	0.0264 (7)	−0.0004 (5)	−0.0177 (6)
F3	0.0501 (7)	0.0913 (11)	0.0491 (7)	0.0241 (7)	−0.0169 (6)	0.0064 (6)
F4	0.0556 (7)	0.0478 (7)	0.0322 (6)	−0.0145 (5)	0.0126 (5)	−0.0010 (5)
O1	0.0181 (6)	0.0575 (8)	0.0364 (7)	−0.0033 (5)	0.0027 (5)	−0.0081 (6)
N1	0.0184 (7)	0.0400 (8)	0.0262 (7)	−0.0022 (6)	0.0015 (5)	−0.0031 (6)
C1	0.0348 (10)	0.0474 (11)	0.0380 (10)	0.0129 (8)	−0.0050 (7)	−0.0027 (8)
C2	0.0310 (9)	0.0411 (10)	0.0291 (8)	−0.0003 (7)	−0.0025 (7)	−0.0016 (7)
C3	0.0221 (8)	0.0419 (10)	0.0343 (9)	0.0000 (7)	−0.0020 (6)	−0.0053 (7)
C4	0.0213 (8)	0.0515 (11)	0.0431 (10)	−0.0013 (7)	0.0015 (7)	−0.0175 (8)
C5	0.0177 (7)	0.0528 (11)	0.0319 (9)	−0.0017 (7)	0.0010 (6)	−0.0128 (8)
C6	0.0230 (8)	0.0756 (14)	0.0303 (9)	0.0149 (8)	−0.0067 (7)	−0.0193 (9)
C7	0.0487 (12)	0.0853 (17)	0.0305 (10)	0.0309 (12)	−0.0013 (8)	0.0013 (10)
C8	0.0299 (10)	0.125 (2)	0.0555 (13)	0.0254 (12)	−0.0168 (9)	−0.0512 (15)
C9	0.0204 (8)	0.0405 (9)	0.0236 (8)	0.0000 (6)	−0.0023 (6)	0.0007 (7)
C10	0.0243 (8)	0.0331 (9)	0.0279 (8)	0.0008 (6)	−0.0030 (6)	−0.0003 (6)
C11	0.0318 (9)	0.0462 (11)	0.0456 (10)	−0.0094 (8)	0.0050 (8)	−0.0067 (9)
C12	0.0365 (11)	0.0575 (13)	0.0714 (15)	−0.0153 (9)	−0.0001 (10)	−0.0182 (11)
C13	0.0454 (12)	0.0618 (15)	0.0575 (14)	−0.0002 (10)	−0.0171 (10)	−0.0237 (11)
C14	0.0522 (12)	0.0496 (12)	0.0309 (10)	0.0064 (9)	−0.0087 (8)	−0.0076 (8)
C15	0.0336 (9)	0.0323 (9)	0.0312 (8)	0.0005 (7)	−0.0022 (7)	0.0018 (7)

### Geometric parameters (Å, °)

**Table:**

Cl1—C2	1.7291 (19)	C6—C7	1.501 (3)
F1—C1	1.334 (2)	C6—C8	1.520 (3)
F2—C1	1.336 (2)	C7—H7A	0.9600
F3—C1	1.337 (2)	C7—H7B	0.9600
F4—C15	1.357 (2)	C7—H7C	0.9600
O1—C9	1.2265 (19)	C8—H8A	0.9600
N1—C9	1.359 (2)	C8—H8B	0.9600
N1—C10	1.414 (2)	C8—H8C	0.9600
N1—H1	0.84 (2)	C10—C11	1.385 (2)
C1—C2	1.490 (3)	C10—C15	1.387 (2)
C2—C3	1.324 (3)	C11—C12	1.394 (3)
C3—C4	1.472 (2)	C11—H11	0.9300
C3—H3	0.9300	C12—C13	1.381 (3)
C4—C6	1.510 (3)	C12—H12	0.9300
C4—C5	1.542 (2)	C13—C14	1.374 (3)
C4—H4	0.9800	C13—H13	0.9300
C5—C9	1.486 (2)	C14—C15	1.376 (2)
C5—C6	1.516 (2)	C14—H14	0.9300
C5—H5	0.9800		
			
C9—N1—C10	125.07 (14)	C6—C7—H7A	109.5
C9—N1—H1	118.4 (13)	C6—C7—H7B	109.5
C10—N1—H1	116.2 (13)	H7A—C7—H7B	109.5
F1—C1—F2	106.39 (16)	C6—C7—H7C	109.5
F1—C1—F3	106.75 (16)	H7A—C7—H7C	109.5
F2—C1—F3	106.13 (15)	H7B—C7—H7C	109.5
F1—C1—C2	111.95 (15)	C6—C8—H8A	109.5
F2—C1—C2	112.92 (15)	C6—C8—H8B	109.5
F3—C1—C2	112.24 (16)	H8A—C8—H8B	109.5
C3—C2—C1	123.42 (17)	C6—C8—H8C	109.5
C3—C2—Cl1	123.62 (14)	H8A—C8—H8C	109.5
C1—C2—Cl1	112.96 (14)	H8B—C8—H8C	109.5
C2—C3—C4	124.87 (17)	O1—C9—N1	122.69 (15)
C2—C3—H3	117.6	O1—C9—C5	124.10 (14)
C4—C3—H3	117.6	N1—C9—C5	113.21 (13)
C3—C4—C6	122.04 (17)	C11—C10—C15	117.50 (16)
C3—C4—C5	121.17 (15)	C11—C10—N1	124.06 (15)
C6—C4—C5	59.59 (12)	C15—C10—N1	118.45 (15)
C3—C4—H4	114.4	C10—C11—C12	119.93 (18)
C6—C4—H4	114.4	C10—C11—H11	120.0
C5—C4—H4	114.4	C12—C11—H11	120.0
C9—C5—C6	122.70 (16)	C13—C12—C11	120.8 (2)
C9—C5—C4	120.83 (14)	C13—C12—H12	119.6
C6—C5—C4	59.16 (12)	C11—C12—H12	119.6
C9—C5—H5	114.4	C14—C13—C12	120.01 (19)
C6—C5—H5	114.4	C14—C13—H13	120.0
C4—C5—H5	114.4	C12—C13—H13	120.0
C7—C6—C4	121.00 (15)	C13—C14—C15	118.51 (18)
C7—C6—C5	120.16 (17)	C13—C14—H14	120.7
C4—C6—C5	61.25 (11)	C15—C14—H14	120.7
C7—C6—C8	114.65 (19)	F4—C15—C14	119.13 (16)
C4—C6—C8	115.1 (2)	F4—C15—C10	117.64 (14)
C5—C6—C8	114.35 (17)	C14—C15—C10	123.23 (17)
			
F1—C1—C2—C3	0.4 (2)	C9—C5—C6—C8	−144.5 (2)
F2—C1—C2—C3	−119.7 (2)	C4—C5—C6—C8	106.4 (2)
F3—C1—C2—C3	120.38 (19)	C10—N1—C9—O1	−6.9 (3)
F1—C1—C2—Cl1	−179.35 (13)	C10—N1—C9—C5	173.01 (15)
F2—C1—C2—Cl1	60.60 (19)	C6—C5—C9—O1	−51.2 (3)
F3—C1—C2—Cl1	−59.32 (19)	C4—C5—C9—O1	19.8 (3)
C1—C2—C3—C4	−179.00 (16)	C6—C5—C9—N1	128.98 (16)
Cl1—C2—C3—C4	0.7 (3)	C4—C5—C9—N1	−160.06 (16)
C2—C3—C4—C6	−156.12 (17)	C9—N1—C10—C11	36.9 (3)
C2—C3—C4—C5	132.50 (19)	C9—N1—C10—C15	−143.37 (17)
C3—C4—C5—C9	−0.8 (3)	C15—C10—C11—C12	1.5 (3)
C6—C4—C5—C9	−112.11 (19)	N1—C10—C11—C12	−178.77 (18)
C3—C4—C5—C6	111.3 (2)	C10—C11—C12—C13	−0.9 (3)
C3—C4—C6—C7	−0.1 (3)	C11—C12—C13—C14	−0.2 (4)
C5—C4—C6—C7	109.82 (19)	C12—C13—C14—C15	0.6 (3)
C3—C4—C6—C5	−109.92 (18)	C13—C14—C15—F4	−179.68 (19)
C3—C4—C6—C8	144.87 (17)	C13—C14—C15—C10	0.2 (3)
C5—C4—C6—C8	−105.21 (17)	C11—C10—C15—F4	178.64 (16)
C9—C5—C6—C7	−2.1 (3)	N1—C10—C15—F4	−1.1 (2)
C4—C5—C6—C7	−111.14 (18)	C11—C10—C15—C14	−1.2 (3)
C9—C5—C6—C4	109.03 (18)	N1—C10—C15—C14	179.05 (17)

### Hydrogen-bond geometry (Å, °)

**Table:**

*D*—H···*A*	*D*—H	H···*A*	*D*···*A*	*D*—H···*A*
N1—H1···O1^i^	0.84 (2)	2.13 (2)	2.9549 (18)	167.3 (18)

Symmetry codes: (i) *x*−1/2, *y*, −*z*+3/2.
